# Protection of Bevacizumab From Silicone Oil–Induced Inactivation by Liposomal Encapsulation

**DOI:** 10.1167/tvst.15.10.27

**Published:** 2026-10-19

**Authors:** Sabrina Wohlfart, Niklas Junker, Hellen Gertrud Röttgen, Manuel Ben Böhmann, Victor Aristide Augustin, Gerd Uwe Auffarth, Philipp Uhl, Maximilian Hammer

**Affiliations:** 1 David J Apple Laboratory for Vision Research, University Eye Clinic Heidelberg, Heidelberg, Germany; 2 Department of Nuclear Medicine, Heidelberg University Hospital, Heidelberg, Germany; 3 Department of Ophthalmology, University Clinic Heidelberg, Heidelberg, Germany; 4 Institute of Pharmacy and Molecular Biotechnology, University of Heidelberg, Heidelberg, Germany

**Keywords:** liposomes, antibody drugs, bevacizumab, drug delivery

## Abstract

**Purpose:**

Silicone oil endotamponades in retinal surgery induce aggregation and thus deactivate antibody drug formulations. Liposomes represent an approach to protect and deliver these drugs when administered intravitreally; however, their interactions with silicone oil endotamponades and their influence on encapsulated antibody agents have never been investigated.

**Methods:**

Bevacizumab was encapsulated in liposomes using dual centrifugation. Size, polydispersity index, zeta potential, and ultrastructure were characterized using dynamic light scattering, laser Doppler electrophoresis, and electron microscopy prior to and after incubation with Siluron 1000 and Densiron Xtra for up to 4 weeks. The behavior of liposomal bevacizumab was assessed using fluorescent ATTO 488 labeling. The size and circular dichroism of bevacizumab were measured after exposure to silicone oil and lysis of the liposomes.

**Results:**

Liposomal formulations of bevacizumab were stable upon silicone oil exposure for up to 4 weeks and showed no signs of lysis or migration. Encapsulated bevacizumab remained solely intraliposomal and did not diffuse through the lipid bilayer, as tracked by fluorescence measurements. The secondary structure of the antibody was preserved throughout the study, as demonstrated by circular dichroism. In contrast to non-formulated bevacizumab, liposomal bevacizumab showed no signs of aggregation after silicone oil exposure.

**Conclusions:**

The liposomal encapsulation of antibodies is an effective method to prevent their silicone oil–induced aggregation and inactivation. Liposomes are resistant to silicone oil exposure for a clinically relevant duration. Further in vivo studies should elucidate efficacy and release kinetics of liposomal encapsulated antibody drugs.

**Translational Relevance:**

This study unravels the potential of liposomes for application in silicone oil–filled eyes.

## Introduction

The intravitreal application of drugs targeting vascular endothelial growth factor (VEGF) plays an important role in vascular retinal diseases.^1^ All currently approved therapeutics are antibodies or fragments of such.^1^^,^^2^ In severe cases, such as proliferative diabetic retinopathy with tractional retinal detachments, vitreoretinal surgery may become necessary in eyes undergoing intravitreal injections of anti-VEGF agents. These cases may require the usage of silicone oils, a lipophilic long-term endotamponade consisting of 100% polydimethysiloxane (PDMS).^3^^–^^6^ Although the surgical,^7^^,^^8^ physicochemical,^9^ and pharmacological^10^ properties of silicone oils have been extensively studied and majorly improved over the last years, most recent investigations have reported that silicone oils deactivate commonly used antibody formulations, such as anti-VEGF drugs, by inducing aggregation.^11^ This property places substantial limitations on the concomitant use of antibody-based anti-VEGF drugs in surgically treated eyes filled with a silicone oil endotamponade, in addition to compromising the group of eyes requiring pharmacological intervention the most.

Among different innovative drug-delivery systems,^12^^,^^13^ liposomes, nanoparticles with a hydrophilic core enclosed by one or more lipid-based bilayers,^14^ represent a promising approach to protect antibody drugs; they have been established and approved for cancer therapy, antifungal therapy, and vaccination^15^ for decades. Figure 1 shows the proposed mechanism of antibody aggregation upon silicone oil exposure and the possible protective effect of liposomal encapsulation. However, the clinical use of liposomal formulations in ophthalmology is so far limited to topical application for the treatment of dry-eye syndrome,^16^^,^^17^ although previous animal studies have suggested the potential of liposomal encapsulation in intravitreal pharmacotherapy.^18^^–^^20^ Moreover, the interaction between liposomes and silicone oil endotamponades, as well as the influence of liposomal formulations on the secondary structure and aggregation of antibody agents, has never been studied.

**Figure 1.**
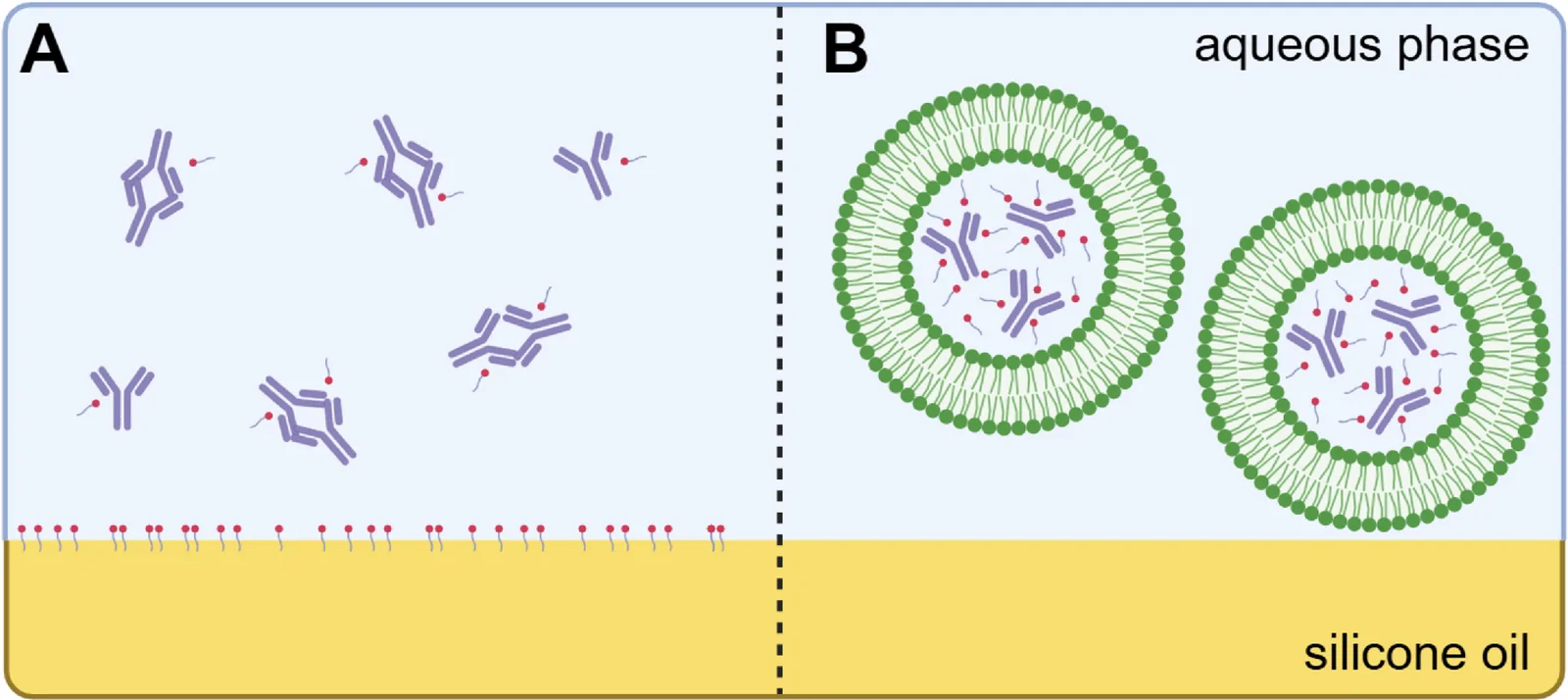
Silicone oil induced aggregation of antibody drugs and liposomal protection. In commonly applied formulations of antibody drugs, surfactants such as polysorbates are added to prevent the antibodies from spontaneous aggregation and inactivation. (**A**) When exposed to silicone oils, these amphiphilic surfactants concentrate on the silicone oil–water interface, effectively reducing the concentration in the aqueous phase and promoting antibody aggregation.^11^ (**B**) Liposomes are an established method to encapsulate drugs within a stable spherical nanostructure to protect them from interactions with the surrounding environment and were investigated as carrier systems for antibody drugs in this study.

This study aimed to evaluate the interaction of liposomally formulated antibody drugs with silicone oil endotamponades for the first time. Therefore, Bevacizumab was encapsulated in liposomes and exposed in a previously described posterior-segment model^10^^,^^21^ to two commonly used silicone oils: the lighter-than-water silicone oil Siluron 1000 and Densitron Xtra, a heavier-than-water silicone oil (both Fluoron GmbH, Ulm, Germany). Densiron Xtra is a heavier-than-water silicone oil, the use of which has been previously described in a posterior-segment model^10^^,^^21^ for up to 4 weeks. Rhodamine labeling of the liposomes was used to assess their migration into the silicone oil, and the stability of the liposomes was evaluated via measurement of the particle characteristics. Localization and intraliposomal concentrations of encapsulated bevacizumab were investigated using fluorescence measurements obtained from ATTO labeling (Leica Microsystems, Wetzlar, Germany) of bevacizumab. Further, the secondary structure of bevacizumab and antibody aggregation were investigated after silicone oil exposure and subsequent lysis of the liposomes.

## Methods

### Materials

Siluron 1000 (S1000) and Densiron Xtra (DXtra) were provided by Fluoron GmbH. Water plus 0.1% formic acid (mass spectrometry grade) and acetonitrile (mass spectrometry grade) were purchased from Carl Roth (Karlsruhe, Germany). Soy phosphosphatidylcholine (SPC) was provided by Lipoid GmbH (Ludwigshafen, Germany). SiLibeads were purchased from Sigmund Lindner (Warmensteinach, Germany); balanced salt solution (BSS), from Beaver-Visitec International (Waltham, MA); Dulbecco's phosphate-buffered saline (DPBS), from Thermo Fisher Scientific (Waltham, MA); and bevacizumab (Zirabev), from Pfizer Europe (Brussels, Belgium). Cholesterol, chloroform, methanol, dimethylsulfoxide, and sodium bicarbonate were purchased from Sigma-Aldrich (St. Louis, MO).

### ATTO 488 Labeling

In a first step, bevacizumab was rebuffered in PBS using an Amicon Ultra Centrifugal Filter (30-kDa molecular weight cut-off [MWCO]; MilliporeSigma, Burlington, MA). Then, 500 µL of bevacizumab stock (25 mg/mL) was added to the centrifugal filter unit and centrifuged for 15 minutes at 14,000*g* at room temperature according to manufacturer's protocol. After centrifugation, 450 µL PBS was added. The entire process was repeated three times. The pH was adjusted to 8.5 with 0.2-M sodium bicarbonate solution. Afterwards, 2 µL ATTO 488 NHS-ester (20 mg/mL in dimethyl sulfoxide [DMSO]; Leica Microsystems) was added, and the solution was incubated for 16 hours at room temperature under gentle rocking. The purification was performed using a NAP-5 column (Cytiva, Marlborough, MA).

The ATTO 488–labeled bevacizumab (bevacizumab–ATTO 488) was further characterized using an Q Exactive Plus Orbitrap mass spectrometer (Thermo Fisher Scientific) with an Agilent 1200 HPLC system (Agilent Technologies, Santa Clara, USA). A MassPREP Micro Desalting Column (2.1 × 5 mm, 20 µm, 1000 Å; Waters, Milford, MA) at 25°C was connected as the stationary phase, and the following gradient was used for separation: 0 to 2 minutes, 100% water (+ 0.1% formic acid); 2 to 2.5 minutes, linear gradient to 50% acetonitrile; 2.5 to 5 minutes, 50% acetonitrile; and 5 to 7 minutes, linear gradient to 100% acetonitrile at a flow rate of 0.5 mL/min. The drug-to-antibody ratio was determined using UniDec software.^22^

### Liposome Preparation

Liposomes were prepared using dual centrifugation. SPC and cholesterol were dissolved in a chloroform/methanol mixture (9:1 v/v) and mixed in a 2-mL screw-cap microtube to achieve a final molar ratio of 90:10 (SPC:cholesterol). The organic solvent was evaporated under a stream of nitrogen at 50°C for 30 minutes. Residual solvent traces were further removed under vacuum in a desiccator for 1 hour at room temperature. To generate sufficient shear forces for a homogeneous size distribution, SiLibeads (type ZY-E, diameter of 1.0–1.2 mm, bulk density of 3.67 kg/L, roundness ≥ 0.96; Sigmund Lindner, Warmensteinach, Germany) were added to the dried lipid film (bead-to-lipid mass ratio of 5:1). Dual centrifugation was performed using a Zentrimix 380 R (Hettich GmbH & Co. KG, Tuttlingen, Germany) at 2500 rpm. The hydration process consisted of two steps. Initially, bevacizumab (25 mg/mL) was added in volumes of 27 µL and 90 µL prior to the first (15 minutes) and second (5 minutes) runs, respectively. Finally, the liposomal dispersion was diluted with an additional 133 µL of BSS and briefly vortexed.

Additionally, rhodamine-labeled liposomes with bevacizumab or bevacizumab–ATTO 488 were synthesized. One part of SPC was replaced by one part rhodamine lipid (18:1; Lissamine Rhodamine PE; Avanti Polar Lipids, Alabaster, AL) to reach a final molar ratio of 89:10:1 (SPC:cholesterol:rhodamine lipid). The rehydration was performed with a solution of bevacizumab or bevacizumab–ATTO 488 in BSS and then centrifuged as described above.

### Liposome Purification

Unencapsulated bevacizumab or bevacizumab–ATTO 488 was removed using a HiTrap Protein A HP antibody purification column (Cytiva) connected to a syringe pump (Landgraf Laborsysteme, Langenhagen, Germany) with a flow rate of 1 mL/min. The purification steps were performed according to the manufacturer's protocol.

### Characterization of the Liposomes

Using dynamic light scattering, we analyzed liposomes for hydrodynamic size, polydispersity index (PDI), and zetapotential, as described before.^18^ In brief, liposomes were diluted to 1 mg/mL lipid compound in 10% DPBS (zeta potential) or 0.1 mg/mL lipid compound in DPBS (hydrodynamic size, PDI) and measured using the automatic mode on the Zetasizer Ultra (Malvern Panalytical, Malvern, UK).

### Quantification of Encapsulation Efficacy and Drug Loading

To determine the encapsulated amount of bevacizumab–ATTO 488, the liposomes were analyzed by fluorescence intensity and compared to a calibration curve of unpurified liposomes of known concentration. Liposomes were dissolved and serially diluted in a 1% aqueous solution of Triton X-100 and analyzed for ATTO 488 and rhodamine using a F200 Pro microplate reader (Tecan, Männedorf, Switzerland) at 485-nm excitation and 535-nm emission (ATTO 488) or at 540-nm excitation and 590-nm emission (rhodamine). Encapsulation efficiency was calculated after normalization for rhodamine and comparison to the amount applied of bevacizumab–ATTO 488 during liposome preparation. Drug loading was calculated by comparing the absolute amount of encapsulated bevacizumab–ATTO 488 to the total lipid amount.

### Determination of Cell Viability

For determination of cell viability, bevacizumab containing liposomes and free bevacizumab as control were tested on ARPE-19 cells using a PrestoBlue assay (Thermo Fisher Scientific). In a 96-well plate, 10^4^ cells per well were seeded and incubated for 24 hours. Then, the medium was removed and the wells were washed with 200 µL PBS. Subsequently, the cells were incubated with the respective treatment (0.08–10 mg/mL liposomes or 0.004–0.5 mg/mL free bevacizumab) in medium for 24 hours. As negative control, Triton X-100 (1% in PBS) was used. Cell medium served as a positive control. In the next step, the treatment was removed, and the wells were washed with 200 µL PBS. Then, PrestoBlue working solution (1:10 in medium) was added followed by incubation for 2 hours. The cell viability was evaluated by measuring the fluorescence at 590 nm with an excitation of 540 nm in a plate reader (Tecan Infinite F200 PRO). The measurement was conducted in triplicates.

### Posterior Segment Model

The posterior segment model used in this study was previously described by our group.^10^^,^^21^ In brief, a tube was filled with 2.25 mL of S1000 or DXtra and 250 µL of the aqueous phase and liposomes loaded with either bevacizumab or bevacizumab–ATTO 488 were injected. Pure bevacizumab served as the baseline control. The tube was then placed on an orbital shaker for 24 hours. The aqueous phase was extracted and further analyzed as previously described.^10^ The experimental setup of this method is depicted in Figure 2.

**Figure 2.**
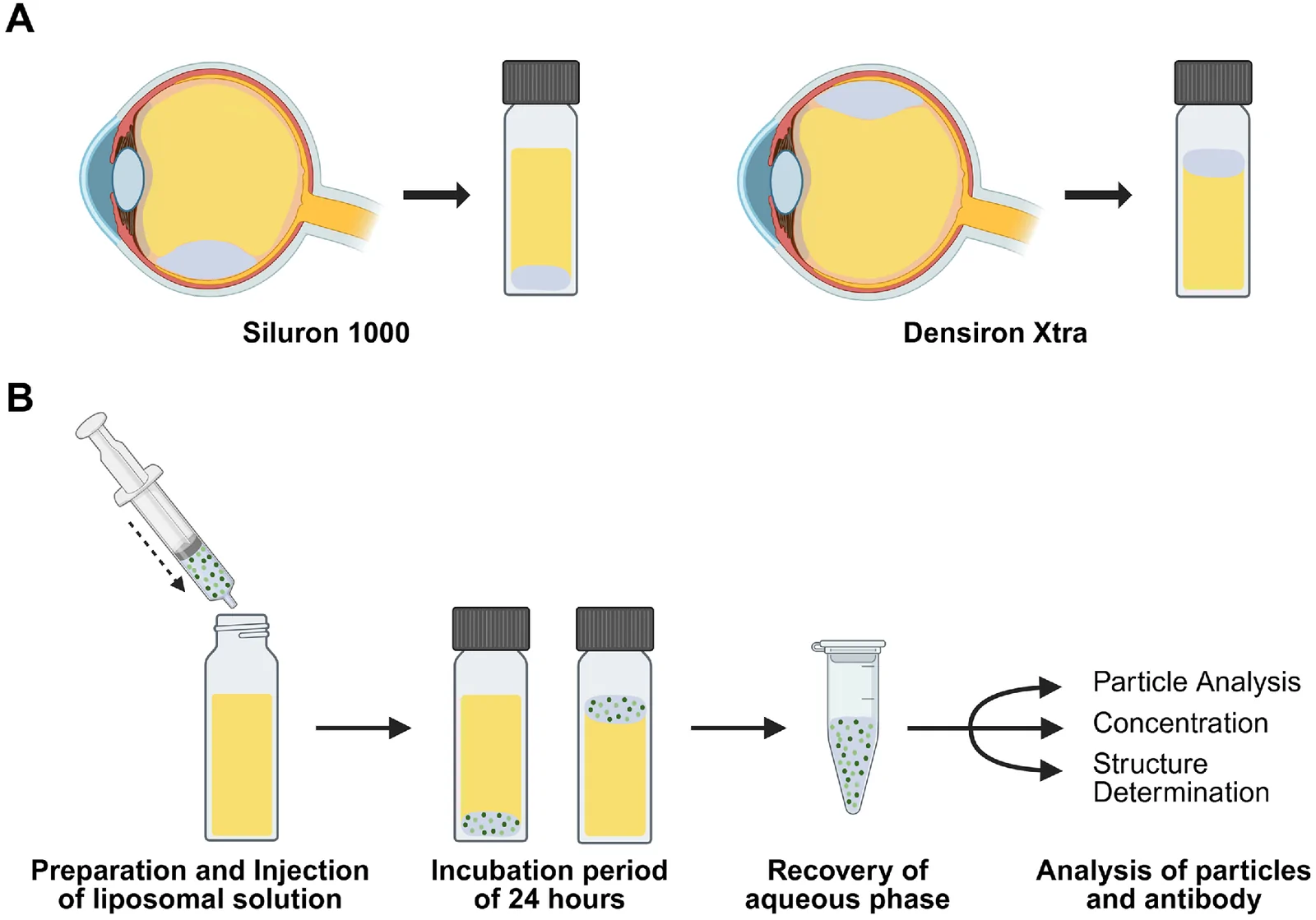
Experimental setup of the posterior segment model. (**A**) The intraocular situation after implantation of lighter-than-water silicone oil or heavier-than-water silicone oil was modeled by a silicone oil–filled tube with an aqueous phase underneath or above the silicone oil phase. (**B**) The prepared liposomes were added to the silicone oil in an aqueous solution and incubated for a study period of 24 hours for up to 7 days. After the incubation, the aqueous phase was recovered, and characteristics and concentrations of the particles, as well as the secondary structure of the antibody, were evaluated.

### In Vitro Quantification of Released Bevacizumab in Silicone Oil

After the incubation, the residual liposomes inside the aqueous phase were recovered and analyzed for the fluorescence intensity of rhodamine and ATTO 488 using the Tecan Infinite F200 PRO microplate reader as described above. To quantify the release of bevacizumab and the migration of liposomes, the obtained values were compared to purified liposomes before silicone oil incubation.

### Cryogenic Transmission Electron Microscopy

Liposomes exposed to S1000 or DXtra, as well as liposomes without exposure to silicone oil, were examined. After application to Holey Carbon–coated grids (Ted Pella, Redding, CA) and initially glow‐discharged for 45 seconds at 20 mA in GloQube Plus (Quorum Technologies, East Sussex, UK), the samples were incubated for 10 seconds at 10°C and 90% humidity. After incubation, blotting of the grids from the back side using Whatman grade 1 filter paper and vitrification in liquid ethane at −180°C with a Leica EM GP2 plunger (Leica Microsystems) was performed. The vitrified grids were transferred to a Talos 200 electron microscope (Thermo Fisher Scientific) using a Gatan 626 Cryo‐Holder (Gatan, Pleasanton, CA), and electron micrographs were acquired at an accelerating voltage of 200 kV using a low‐dose system (40 e– Å^–^^2^). During the imaging process, the temperature of the samples was maintained at −175°C.

### Structural Determination of Bevacizumab

The secondary structure of bevacizumab was evaluated using the measurement of circular dichroism (CD) of the antibody. Prior to measurement of circular dichroism, lysis of the liposomes by dissolution with 5 parts of methanol was necessary. After the particles were dissolved, the clear solution was placed in a quartz cuvette (1 mm; Hellma GmbH & Co. KG, Mühlheim, Germany) and analyzed with a J-1700 CD Spectrometer (JASCO, Easton, MD) in a wavelength range from 190 to 260 nm. Pure bevacizumab with and without contact to silicone oil served as a control.

### Sodium Dodecyl Sulfate–Polyacrylamide Gel Electrophoresis

Non-denaturating sodium dodecyl sulfate–polyacrylamide gel electrophoresis (SDS-PAGE) was performed using a 5% stacking and 8% separating gel in a Mini-PROTEAN Tetra Cell electrophoresis chamber (Bio-Rad, Laboratories, Hercules, CA). Separation was performed with 110 V for 3 hours, and a ProSieve QuadColor Protein Marker, 4.6 to 300 kDa (Lonza, Basel, Switzerland) electrophoresis marker was used.

### Statistical Analysis

Samples were prepared in at least independent triplicates. The number of replicates for each experiment is indicated in the figure description. Sample size planning was performed based on our experience from previous investigations. Repeated-measures ANOVA and ordinary one-way ANOVA with Tukey's or Dunnett's post hoc test were performed as appropriate using Prism 11.0.0 (GraphPad Software, Boston, MA).

## Results

### Particle Characteristics

Liposomal formulation of bevacizumab was successful with mean ± SD encapsulation efficiency of 4.25% ± 0.15% and a drug load of 1.99% ± 0.09%/mg. The resulting liposomes showed an average size of 108.6 ± 3.8 nm with a PDI of 0.21 ± 0.01 and a zeta potential of –11.9 ± 2.2 mV when containing bevacizumab and an average size of 97.2 ± 2.5 nm with a PDI of 0.17 ± 0.02 and a zeta potential of –11.2 ± 1.0 mV when containing bevacizumab–ATTO 488 (Fig. 3A).

**Figure 3.**
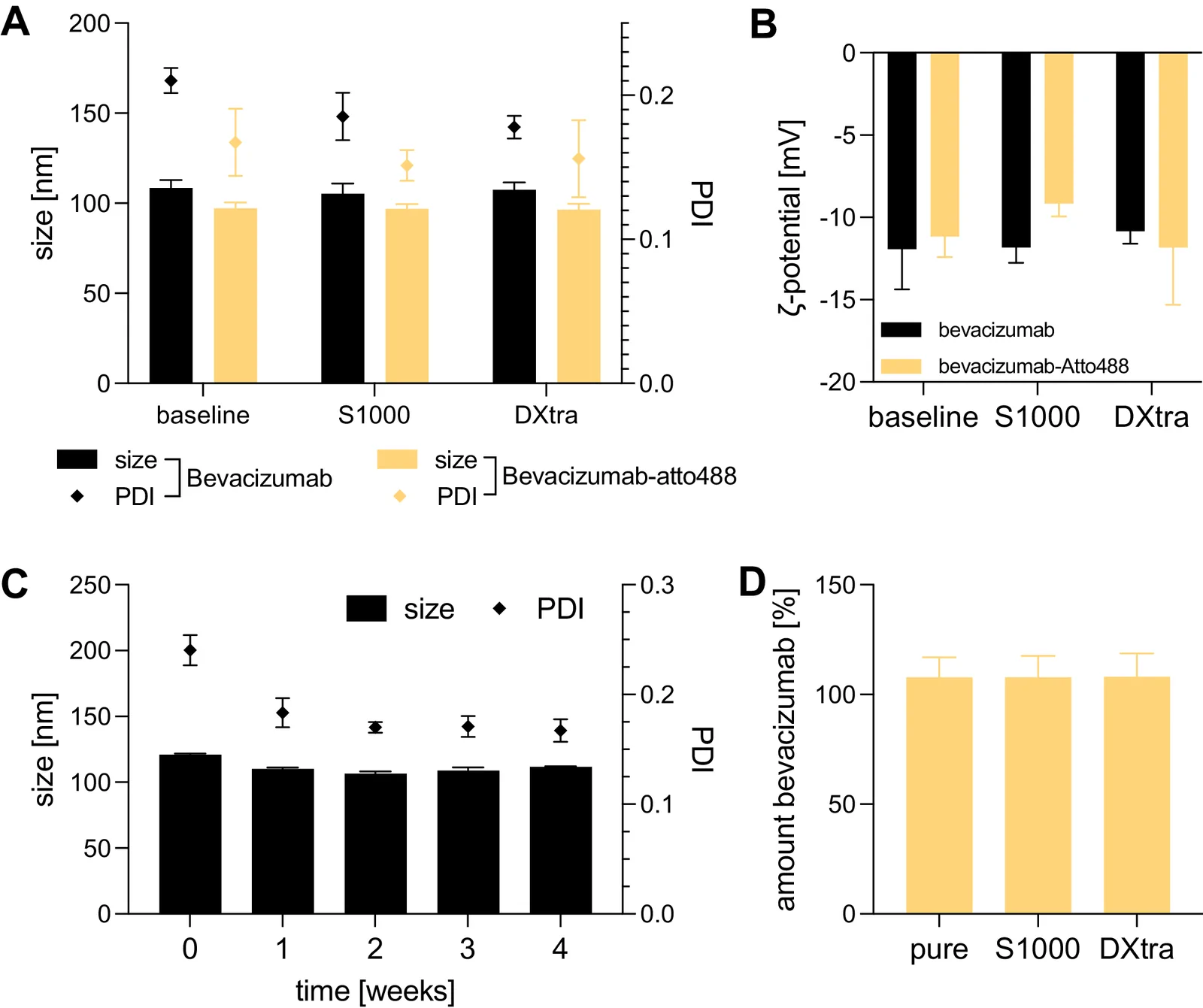
Overview of the particle characteristics in the aqueous phase after silicone oil exposure. (**A, B**) Particle size and PDI (**A**) and zeta potential (**B**) of liposomes containing bevacizumab (*n* = 6, *black*) or bevacizumab–ATTO 488 (*n* = 9, *yellow*) showed no relevant changes after incubation in lighter-than-water S1000 and heavier-than-water DXtra compared to baseline. (**C**) During an extended incubation period of 4 weeks in S1000, both size and PDI remained stable for liposomes containing bevacizumab (*n* = 3). (**D**) The amount of ATTO 488–labeled bevacizumab within the liposomes was the same before and after exposure to S1000 or DXtra for 24 hours, indicating no spontaneous lysis of the liposomes upon silicone oil exposure (*n* = 3). All measurements are shown as mean ± SD.

### Liposomal Encapsulated Bevacizumab Showed No Cytotoxic Effects on ARPE-19 Cells

Growth and viability of ARPE-19 cells were not impaired by either liposomal encapsulated or free bevacizumab in concentrations between 0.08 and 10 mg/mL or 0.004 to 0.5 mg/mL, respectively, indicating an excellent cellular biocompatibility (Supplementary Fig. S1).

### Particle Characteristics and Bevacizumab Loading Are Stable After Silicone Oil Exposure

After incubation of both particle types in heavier-than-water (DXtra) and lighter-than-water (S1000) silicone oil for 24 hours, the particle characteristics size, PDI, and zeta potential remained stable for all samples, and no relevant changes to baseline occurred (Figs. 3A, 3B). After a prolonged incubation period with S1000 of up to 4 weeks, particle size and PDI were quantified at days 1, 2, 3, 7, 14, 21, and 28. After recovery from the aqueous phase, there were no substantial alterations in particle characteristics over the course of the incubation (Fig. 3C). For the liposomes containing bevacizumab–ATTO 488, the measured amount of bevacizumab inside the liposomes was the same before and after silicone oil contact (Fig. 3D).

### Particles Remain in the Aqueous Phase and Do Not Migrate Into the Silicone Oil

During incubation of the liposomal formulations in the aqueous phase with the silicone oils, a distinct border became visible and remained stable over the course of at least 24 hours with no apparent dissolution in the silicone oil phase (Figs. 4A, 4B). In the aqueous phase, the liposomes concentrated at the ground, especially noticeable for the liposomes containing ATTO 488–labeled bevacizumab (Fig. 4A, arrow). Additionally, after recovering the aqueous phase containing rhodamine-labeled liposomes or liposomes containing ATTO 488–labeled bevacizumab liposomes, the amount of fluorescently labeled component was measured. This revealed that the same amount of liposomes remained in the aqueous phase before and after oil contact, so the indissolubility in silicone oil was confirmed (Fig. 4C). The number of liposomes in the aqueous phase before and after incubation with silicone oil remained unchanged, indicating no significant spontaneous lysis or migration of the liposomes into the silicone oil phase (Fig. 4C).

**Figure 4.**
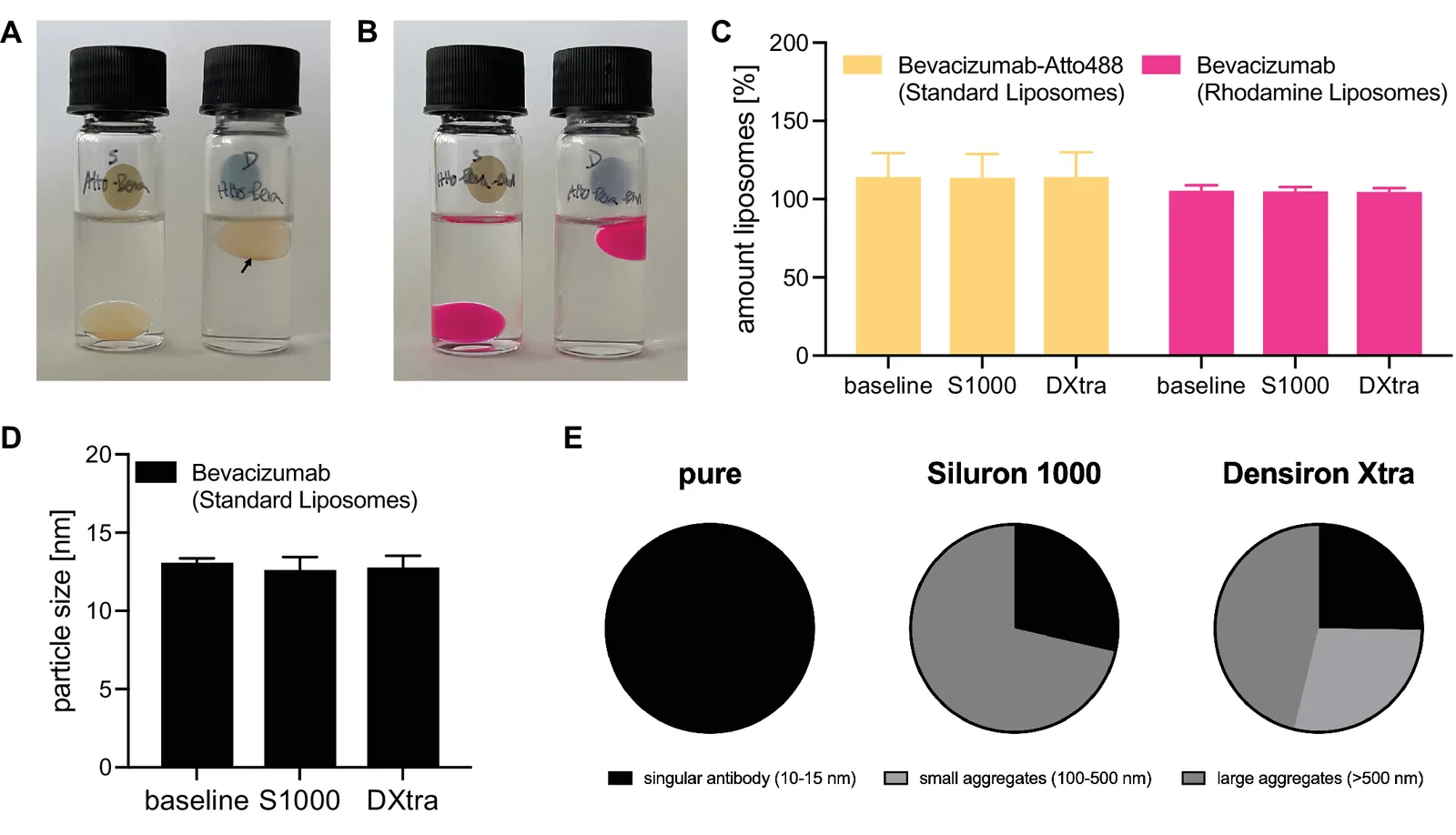
Liposomal stability upon exposure to silicone oil and protection from silicone oil–induced aggregation. (A, B) The liposomes containing bevacizumab–ATTO 488 (**A**) and the rhodamine-labeled liposomes containing unmodified bevacizumab (**B**) showed no signs of spontaneous lysis or dissolution into the lighter-than-water silicone oil (*left*) or the heavier-than-water silicone oil (*right*). Within the aqueous phase, the liposomes concentrated at the bottom (*arrow marking*). After recovery of the aqueous phase and determination of the size and PDI of the liposomes, no non-specific particle aggregation occurred. (**C**) The stability of the liposomes upon silicone oil exposure was further indicated by quantification of the amount of liposomes in the aqueous phase before (baseline) and after exposure to S1000 or DXtra for 24 hours which showed no significant changes (*n* = 3). (**D**) After lysis of the liposomes at the end of the silicone oil exposure, the size of the antibody particles was the same as before the exposure, indicating no presence of silicone oil–induced aggregation of the bevacizumab antibodies with liposomal encapsulation (*n* = 6). (**E**) Without liposomal encapsulation, bevacizumab formed aggregates of different sizes upon silicone oil exposure, as previously described.^11^ All measurements are shown as mean ± SD.

### Intra-Liposomal Bevacizumab Is Protected From Silicone Oil–Induced Aggregation

After lysis of the liposomes at the end of the experiment, a particle size of 11.6 ± 0.4 nm, typical for monoclonal antibodies (e.g., 12.9 ± 0.3 for bevacizumab stock solution), was measured, indicating the protection of bevacizumab from silicone oil–induced aggregation by the liposomal formulation (Fig. 4D). In contrast, bevacizumab without liposomal encapsulation formed aggregates of different size upon exposure to silicone oil (Fig. 4E).

### Liposomal Structure Is Conserved During Contact With Silicone Oil

Cryogenic transmission electron microscopy (TEM) imaging revealed an unimpaired spherical structure of the particles with an intact lipid bilayer envelope after exposure to both S1000 (Fig. 5B) and DXtra (Fig. 5C). No morphological changes appeared when compared to baseline (Fig. 5A), as quantitatively supported by analysis of particle characteristics prior to and after silicone oil exposure (Fig. 3).

**Figure 5.**
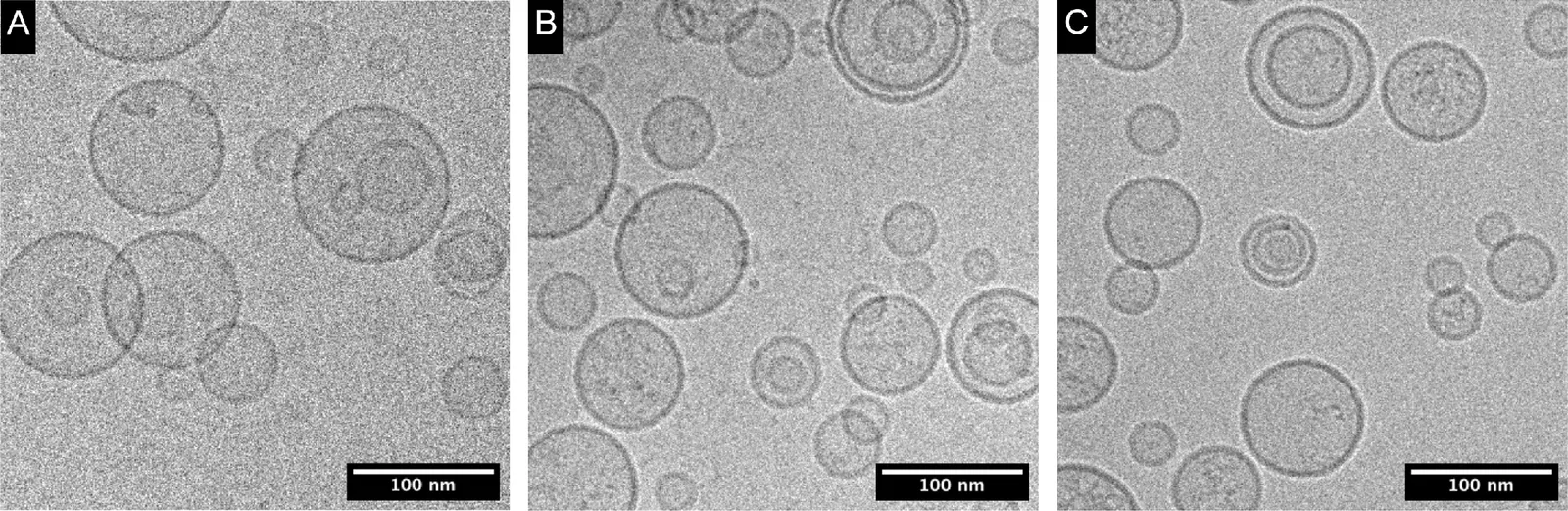
Ultrastructural examination of the liposomes after silicone oil exposure. (**A**) Before contact to the silicone oils, the liposomes were spherical particles about 50 to 100 nm in size, enveloped by a lipid bilayer. (**B**, **C**) After exposure to S1000 (**B**) and DXtra (**C**), the spherical structure of the particles and their size remained stable. There was no impairment of the lipid envelope or signs of spontaneous lysis. Representative cryogenic TEM images are shown.

### Secondary Structure of Encapsulated Bevacizumab Is Preserved After Silicone Oil Exposure

To evaluate the influence of liposomal encapsulation and subsequent exposure to silicone oil on the secondary structure of the antibody, the circular dichroism was determined after solubilization of the liposomes. Typical beta sheet structures were detected for liposomal bevacizumab exposed to S1000 (Fig. 6E) or DXtra (Fig. 6F) with no significant changes in unexposed bevacizumab (Fig. 6C), indicating the structural preservation of bevacizumab during liposomal encapsulation and silicone oil exposure. Importantly, the process of liposome lysis did not interfere with the measurement protocol (Figs. 6A, 6B, 6D). Additionally, non-denaturing SDS-PAGE showed no changes in protein structure after inclusion in the liposomes and oil contact. No antibody aggregates or smaller fragments were identified (Supplementary Fig. S2).

**Figure 6.**
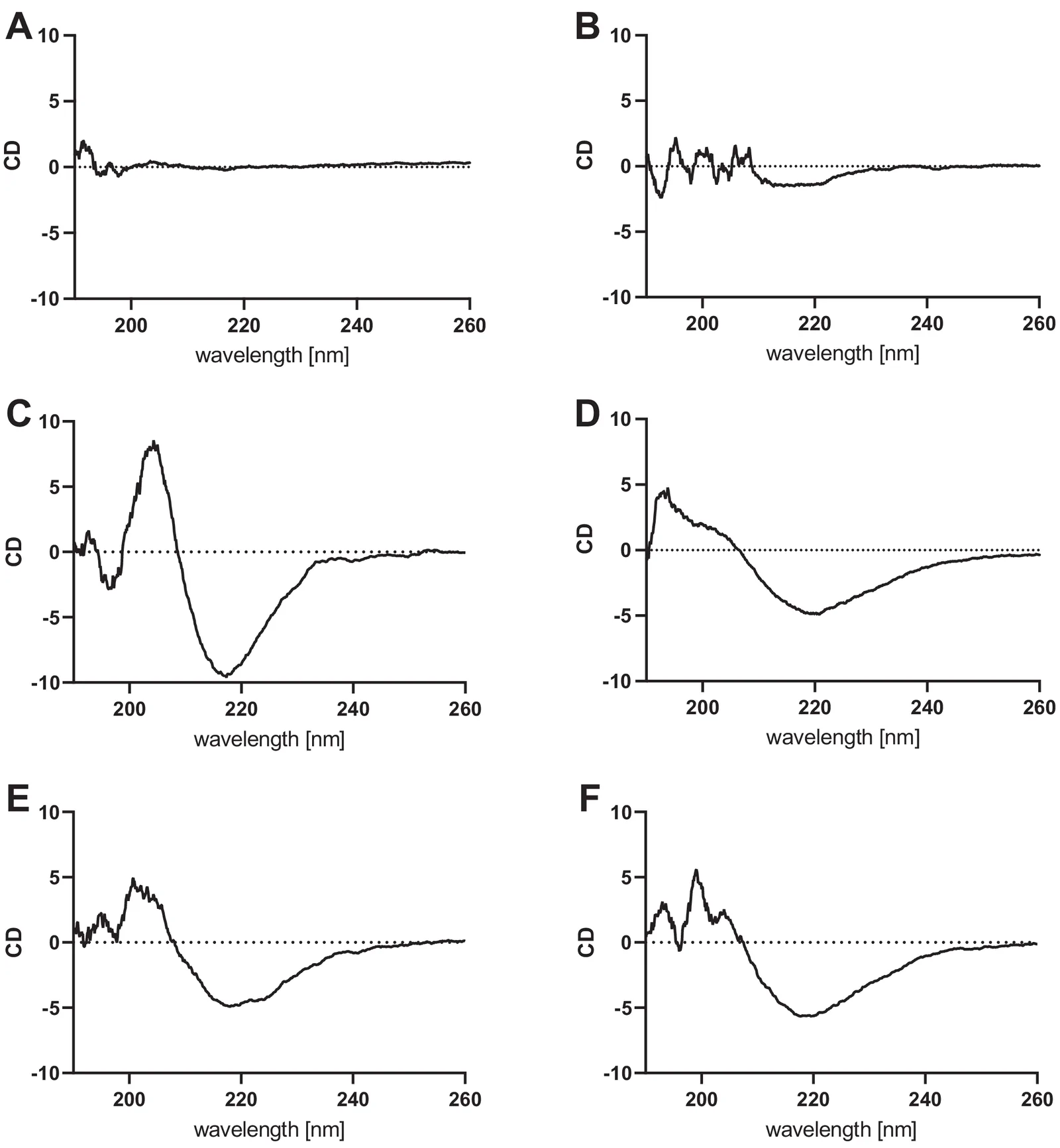
Evaluation of the secondary structure of bevacizumab. Measurement of CD was performed after lysis of the liposomes. (**A**) A lysis solution with a 1:5 BSS-to-methanol ratio served as a blank. (**B**) Undissolved liposomes in BSS showed an unspecific signal. (**C**) Bevacizumab 250 µg/mL in BSS showed the typical beta-sheet structure and served as a control. (**D**) Measurement of CD was not impaired by the lysis procedure, as indicated by an unimpaired CD signal of bevacizumab dissolved in 1:5 BSS-to-methanol ratio. (**E, F**) Bevacizumab after incubation as a liposomal formulation for 24 hours in S1000 (**E**) or DXtra (**F**) and lysis of the liposomes showed no significantly changed CD signal compared to the control, indicating a preservation of the secondary structure through liposomal encapsulation. Individual curves are depicted for one replicate.

## Discussion

This study investigated the general interaction between liposomal formulation and silicone oil for the first time, to our knowledge. We found that liposomes did not dissolve within a silicone oil environment for exposure times of up to 4 weeks in an in vitro posterior segment model, as shown by rhodamine labeling of liposomes, measurement of particle characteristics, and cryogenic TEM imaging. Further, the liposomally formulated antibody drug bevacizumab showed no leakage throughout the entire study period and no signs of diffusion through the lipid bilayer, as evidenced by ATTO 488 labeling. Finally, we characterized the liposomal encapsulation to preserve the secondary structure of antibody drugs such as a bevacizumab and to protect them from their aggregation and inactivation commonly occurring due to silicone oil exposure.

The inactivation of antibody drugs by induction of aggregation driven by silicone oil exposure was described in previous work.^11^ The process of antibody aggregation in this setting is considered complex and influenced by multiple factors, including the accumulation of so-called surfactants on the water–oil interface as one potential mechanism.^11^ Surfactants are ingredients such as polysorbates that are included in antibody formulations to prevent spontaneous aggregation.^23^ Due to their amphiphilic nature, they may concentrate on the interface between a hydrophilic and a hydrophobic phase, leading to a relative lowering of the surfactant concentration within the aqueous phase. This surfactant accumulation may then promote aggregation and inactivation of the antibodies (Fig. 1A).^11^ Liposomes are phospholipid-based, spherical nanoparticles consisting of a hydrophilic core encapsulated by a lipid bilayer.^14^ The lipid bilayer self-assembles through interaction of the hydrophobic parts of the amphiphilic phospholipids with the hydrophilic parts presented on the outside and inside surfaces of the liposome, forming a thermodynamically stable, energetically favorable state.^14^ This stable lipid bilayer structure is likely to be the predominant factor preventing the liposomes from spontaneous solubilization within both hydrophilic and hydrophobic environments and may protect the cargo inside the hydrophilic core from interactions with the surrounding media. In the context of liposomal encapsulation of bevacizumab, liposomes could specifically prevent contact between the protein drug and the silicone oil and thereby seem to prevent destabilization. Further, the potential stabilization could be traced back to the amphiphilic surfactants, also located in the inner aqueous phase of the liposomes, which are also potentially prevented from accumulating on the water–silicone oil interface (Fig. 1B). Additionally, the structural preservation of bevacizumab identified by circular dichroism and SDS-PAGE also indicates no emergence of protein degradation products or changed immunogenic properties, which may impair biocompatibility and translational potential in the future. The protective effect of liposomal formulation was demonstrated in this study only for bevacizumab as a proof of principle; however, the results of this study are believed to be applicable to other antibody-based formulations. The pivotal findings of this study—the stability of liposomal formulation upon silicone oil exposure and its protective effect on the structural integrity of the antibody—is thought to apply regardless of the specific structure of the antibody: When they have been encapsulated, they are protected against silicone oils. Further, as the effect of silicone oil–induced aggregation was shown across different antibodies in previous work,^11^ the findings of this study are therefore hold to be relevant for numerous intravitreally applied antibody drugs.

Aside from their protective effect on the silicone oil–induced aggregation of antibody drugs, liposomes were previously tested as drug carriers in translational ophthalmic research primarily aimed at prolonging the intravitreal half-life of the respective pharmaceutics. Liposomal formulations of immunosuppressants,^24^ antiviral and antifungal drugs,^19^^,^^25^ and even bevacizumab^20^ have been described in prior studies in rat and rabbit models with promising results regarding intravitreal biocompatibility and pharmacokinetics. Most recently, our group introduced a liposomal formulation of antiproliferative methotrexate that showed excellent biocompatibility and a prolonged intravitreal half-life of the small molecule drug from 3 days to up to 6 weeks in an in vivo porcine model.^18^ This underlines the potential of liposomal drug delivery systems to enhance sustained release of their drug cargo and, with regard to the findings of this study, protect it from interaction with the surrounding environment, as specifically shown for the silicone oil–filled eye.

The aim of this study was to demonstrate the principle of antibody protection through liposomal encapsulation upon silicone oil exposure for the first time. This proof-of-principle investigation used a standard liposomal formulation and therefore considered aspects such as encapsulation efficiency only secondarily; however, future studies targeting clinical application should optimize encapsulation efficiency as previously described.^26^ In contrast, the negative zeta potential of the liposomes prepared in our study is typical for liposomal formulations.^27^ Notably, this may further be beneficial by preventing particle–matrix interactions with possible vitreous remnants in future applications in vitrectomized and silicone oil–filled eyes.

In addition to liposomal formulation, multiple efforts have been made in biomaterial science and nanotechnology over the last several decades to improve intravitreal pharmacotherapy and retinal drug delivery.^28^^–^^30^ The use of liposomes in ophthalmology to date has been limited to topical administration, especially in the dry eye disease.^16^^,^^17^ However, the silicone oil–induced inactivation previously described by our group, as well as the protective effect of liposomal encapsulation observed in this study, demonstrate a high clinical relevance, as antibody–based anti-vasoproliferative drugs and silicone oils are both widely established, key components in the therapeutic regimen of complex retinal diseases.^31^^,^^32^ One striking clinical example is diabetic retinopathy, for which anti-VEGF drugs are frequently used in the treatment of its different entities, specifically the proliferative form (PDR)^33^ and diabetic macular edema.^34^ Especially in cases with PDR, eventually vitreoretinal surgery and implantation of silicone oils becomes necessary due to secondary complications such as vitreous hemorrhage or retinal traction detachment.^6^^,^^35^ In these cases, the safe and effective use of anti-VEGF antibody drugs can be critical for the patient's outcome. Furthermore, the therapeutic efficacy in cases with DR is typically limited due to challenges regarding patient compliance and therapy adherence in this patient collective.^36^ Considering the previous preclinical studies on the prolongation of the intravitreal half-life of drugs by liposomal formulation,^18^^,^^20^^,^^25^ reducing the treatment interval by extending the intravitreal effect duration could therefore further enhance the quality of care for those patients.

However, although silicone oil–induced inactivation of antibody drugs seems to be prevented by liposomal formulations, intravitreal administration of antibody drugs is further challenged by the significant alterations regarding pharmacokinetics in the vitrectomized, silicone oil–filled eye, as recently demonstrated by early distribution studies of radioactively labeled bevacizumab.^37^ Especially when applying hydrophilic drugs, phase separation between the drug containing the hydrophilic phase and the hydrophobic silicone oil phase substantially alters intraocular distribution^37^ and may further impair delivery to the target tissue. Because liposomes in this study were shown to respect this phase separation while not migrating into the silicone oil phase, this challenge remains with regard to clinical application.

Evaluation of the in vivo pharmacokinetic and pharmacodynamic properties of encapsulated liposomal bevacizumab in the silicone oil–filled eye is necessary in the context of future development toward a clinical application. In particular, drug release, intraocular distribution, and retinal bioavailability of such liposomal anti-VEGF formulations remain uncertain. Therefore, additional research in an appropriate large animal disease model is needed to evaluate both the biocompatibility and the efficacy of such liposomal formulations of anti-VEGF antibody drugs in the silicone oil–filled eye.

### Limitations

Some limitations of this study should be considered. First, we tested only the stability and protective effect of the liposomes for an exposure time to the silicone oils of up to 4 weeks, as we expected, from our previous work, that antibody aggregation would occur as early as 24 hours after silicone oil exposure.^11^ Because the treatment interval of common intravitreally applied antibody drugs may exceed 10 weeks, this should be considered when interpreting the results. Second, the in vitro setup could only partially mimic the complex ocular microenvironment. We aimed to simulate eye movements using an orbital shaker, but such aspects as head position, saccadic movements, and vitreous and aqueous humor flow could not be addressed completely. Further, our study setup did not allow the evaluation of possible interactions between the liposomal formulation and a biological environment, necessitating follow-up in vitro cell culture experiments and in vivo animal experiments. Third, this study focused on antibody structure and aggregation, but lysis of the liposomes requires chemical treatment that interferes with the performance of ELISA and cell-based methods, so the VEGF-binding capacity and therapeutic efficacy of the antibody after liposomal encapsulation could not be evaluated. Future studies should therefore elucidate these aspects with appropriate techniques and evaluate in vivo therapeutic efficacy to enable further translation of this approach. Finally, although we demonstrated the stability of the liposomes and their protective effect within the silicone oil environment, the in vitro setting did not allow making any statement regarding the biocompatibility of the formulation or the therapeutic efficacy of the encapsulated antibody drug. To evaluate these aspects, testing in an in vivo disease model is necessary.

## Conclusions

Liposomes show stable characteristics upon exposure to silicone oil, successfully protecting antibody drugs such as bevacizumab from silicone oil–induced aggregation and inactivation and preserving their secondary structure over a period of at least 4 weeks. This highlights the potential of liposomes as nanocarriers in intravitreal pharmacotherapy.

## Supplementary Material

Supplement 1
