# Supplementary material for: Protection of Bevacizumab From Silicone Oil–Induced Inactivation by Liposomal Encapsulation
Source: Transl Vis Sci Technol. 2026 Oct 19;15(10):27. doi: 10.1167/tvst.15.10.27 (PMC13712388; doi:10.1167/tvst.15.10.27)
Supplement: Supplementary file 1 [file tvst-15-10-27_s001.pdf]

Supplementary Material

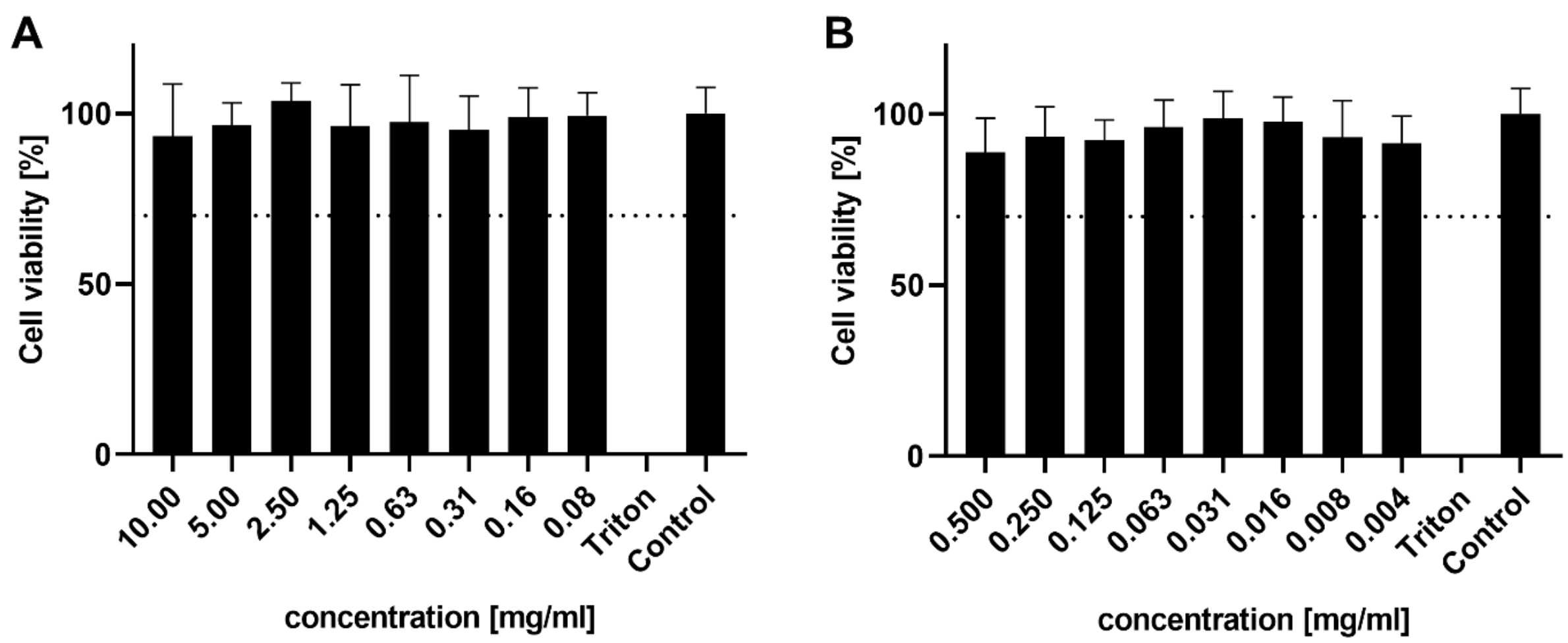

Figure S1. Biocompatibility of bevacizumab and bevacizumab containing liposomes on ARPE-19 cells. Cell viability of ARPE-19 cells using the PrestoBlue™ assay showed no cytotoxic effects for pure bevacizumab (A) and bevacizumab containing liposomes (B). Depicted for all measurements is mean ± SD for N=3 independent replicates.

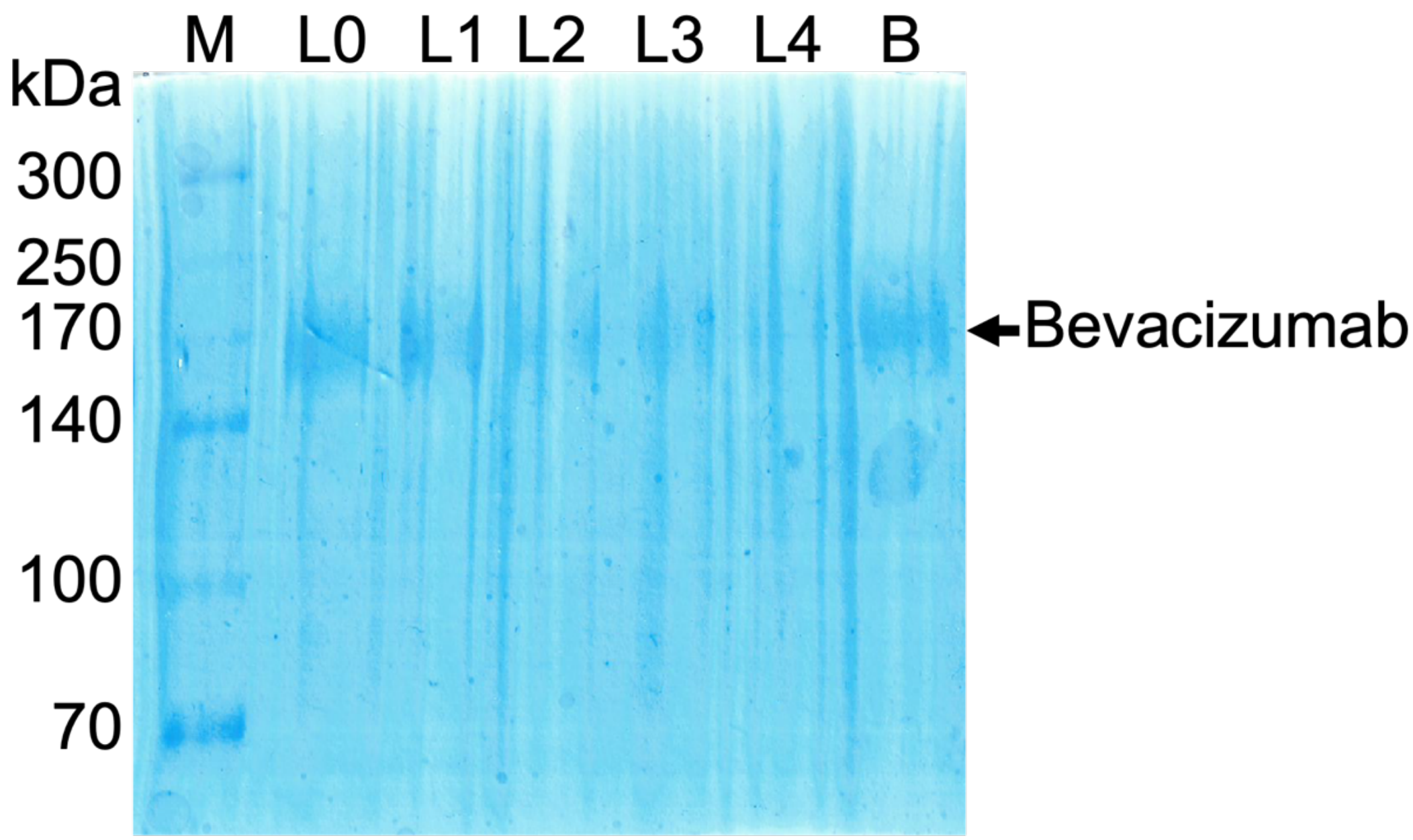

Figure S2. Non-denaturating SDS-Gel shows intact antibody after inclusion in liposomes. M: protein marker, L0: bevacizumab liposomes stock, L1: bevacizumab liposomes after 1 week in Siluron1000, L2: bevacizumab liposomes after 2 weeks in Siluron1000, L3: bevacizumab liposomes after 3 weeks in Siluron1000, L4: bevacizumab liposomes after 4 weeks in Siluron1000, B: bevacizumab stock solution.
